# Supervised Physical Activity Interventions in Children and Adolescents with Cancer Undergoing Treatment—A Systematic Review

**DOI:** 10.3390/curroncol32040234

**Published:** 2025-04-17

**Authors:** Nadja Battanta, Krystyna Lange, Sabine V. Kesting, Daniela Marx-Berger, Philip Heesen, Hannah Ober, Aron Onerup, Saskia M. F. Pluijm, Eva Scheler, Emma J. Verwaaijen, Katrin Scheinemann, Maria Otth

**Affiliations:** 1 Division of Oncology/Hematology, Children’s Hospital of Eastern Switzerland, 9006 St. Gallen, Switzerlandmaria.otth@kispisg.ch (M.O.); 2 Department of Pediatrics, German Center for Child and Adolescent Health (DZKJ), Partner Site Munich, TUM School of Medicine and Health, Technical University of Munich, 80804 Munich, Germany; 3 Institute of Preventive Pediatrics, Department Health and Sport Sciences, TUM School of Medicine and Health, Technical University of Munich, 80804 Munich, Germany; 4 Division of Sportsmedicine, Children’s Hospital of Eastern Switzerland, 9006 St. Gallen, Switzerland; 5 Faculty of Medicine, University of Zurich, 8091 Zurich, Switzerland; 6 Department of Pediatrics, Institute of Clinical Sciences, Sahlgrenska Academy, University of Gothenburg, 40530 Gothenburg, Sweden; 7 Princess Máxima Center for Pediatric Oncology, 3584 CS Utrecht, The Netherlands; 8 Department of Cardiology, Cantonal Hospital St. Gallen, 9007 St. Gallen, Switzerland; 9 Faculty of Health Sciences and Medicine, University of Lucerne, 6005 Lucerne, Switzerland; 10 Department of Oncology, University Children’s Hospital Zurich, 8008 Zurich, Switzerland

**Keywords:** childhood cancer, treatment, exercise intervention, physical activity, quality of life, strength, cardiovascular

## Abstract

*Background:* A cancer diagnosis and its treatment often disrupt a child’s and adolescent’s normal level of physical activity, which plays a vital role in their development and health. They are therefore often less physically active during treatment than before the diagnosis or compared to healthy peers. Today, there is no comprehensive overview of the safety, feasibility, clinical effectiveness, and potentially long-lasting impact of physical activity (PA) interventions in this population. *Methods:* We conducted a systematic review in PubMed according to PRISMA guidelines to evaluate studies on PA interventions during cancer treatment in children and adolescents up to 25 years of age. We used the Joanna Briggs Institute’s critical appraisal tools to assess the risk of bias. Due to the heterogeneity in interventions and outcomes, we used descriptive approaches only to present the results. *Results*: Half of the 21 included studies were randomized controlled trials (10/21). PA interventions were found to be safe and feasible when tailored to the patient’s age, treatment phase, and clinical condition. Most studies reported improvements in physical fitness, strength, and quality of life, with some reductions in fatigue. Variability in interventions and outcomes, along with small sample sizes and heterogeneous patient populations, made it difficult to draw clear conclusions. *Conclusions*: PA appears to be a feasible and, in terms of injuries, safe adjunct to cancer treatment in children and adolescents. Despite promising trends, further large-scale, multicenter trials with standardized protocols are needed to better establish the long-term benefits and optimal interventions.

## 1. Introduction

Childhood cancer remains a significant global health challenge, with over 400,000 new cases diagnosed annually worldwide [1]. Survival has improved dramatically over the last few decades due to improvements in diagnosis, treatment, and supportive care [2]. However, it still is a life-altering diagnosis that affects not only the physical health of young patients but also their emotional well-being, social development and overall quality of life (QoL). The treatment remains challenging, often marked by fatigue, physical deconditioning, and other physical and mental side effects [3,4]. These challenges are compounded by the fact that cancer treatment often disrupts a child’s normal routines, including physical activity, which plays a vital role in their development and health [5,6,7]. The reduction in physical activity and the resulting inactive and sedentary lifestyle can be caused by treatment-related side effects such as fatigue, pain, general malaise, and decreased physical function. Such inactivity may exacerbate side effects and negatively impact physical and mental health. Therefore, interventions that promote physical activity in children and adolescents during treatment may counteract these effects and support their development and QoL [7,8]. These interventions correspond to exercises, equivalent to structured and regular activities as part of overall physical activity.

Current research highlights the potential benefits of physical activity during cancer treatment, even when adapted to the physical limitations imposed by the cancer or its treatment. Therefore, offering physical activity may be an opportunity to support children and adolescents in their recovery and to improve their quality of life. Studies suggest that tailored physical activity programs can help to reduce treatment related fatigue, improve cardiorespiratory fitness and physical functioning, and enhance overall QoL [9,10,11]. While it may seem counterintuitive to encourage activity during such a physically demanding time, carefully monitored exercise programs have been shown to improve physical fitness, muscle strength, and overall well-being in children and adolescents diagnosed with cancer [12,13]. These interventions showed high adherence rates, suggesting that children and their families are willing to participate in structured physical activity programs during treatment [10].

Despite these promising findings, the role of physical activity during cancer treatment in children and adolescents remains underexplored. There is limited consensus on which type, duration, and intensity of activities are effective, the optimal intervention designs, the outcomes to be measured, safety parameters, and feasibility across diverse patient populations.

This systematic review aims to (1) assess the impact of physical activity interventions on physical, psychological, and functional outcomes in children and adolescents treated for cancer, (2) to identify barriers and facilitators to implement physical activity interventions in pediatric oncology settings, (3) to determine the safety and feasibility of physical activity interventions, and (4) to provide inputs for future research to optimize the integration of physical activity into standard cancer care protocols for children.

## 2. Materials and Methods

### 2.1. Eligibility Criteria

This systematic review was conducted in accordance with the Preferred Reporting Items for Systematic Reviews and Meta-Analyses (PRISMA) guidelines [14]. Inclusion criteria were defined using the PICO framework: (P) The population of interest comprised children, adolescents, and young adults diagnosed with any type of cancer up to the age of 25 years and still under treatment at initiation of the intervention. (I) The intervention was defined as any physical activity intervention (e.g., aerobic, stretching, yoga, and/or exergaming). (C) Comparator were given by the original study and included cancer patients without intervention, own comparison by pre and post intervention, active control groups with different activities than the intervention group, or historical cohorts. (O) The primary outcomes were the impact of physical activity interventions on physical and functional outcomes. The secondary outcomes were the impact of physical activity interventions on mental health, QoL, and possible adverse events of the interventions. Eligible study designs had to include comparators (e.g., randomized controlled trials (RCTs), controlled clinical trials (CCTs), cross-over, cluster randomized trial).

### 2.2. Information Sources and Search Strategy

A comprehensive search was conducted across PubMed/MEDLINE, including studies published between January 2000 and May 2024. An additional evidence search was performed using reference screening of identified (systematic) reviews and guidelines/recommendations. The search strategy combined subject headings (e.g., MeSH terms) and keywords of the following concepts: “children”, “cancer”, “physical activity/exercise training/therapy”, and “during treatment” (Supplementary Table S1).

### 2.3. Study Selection and Data Extraction

Titles and abstracts were screened independently by two reviewers using predefined eligibility criteria. Potential publications from the reference screening were included in the title and abstract screening too. Full-text articles were reviewed for studies that met the eligibility criteria. Discrepancies between reviewers were resolved by a third reviewer. The study selection process was documented using a PRISMA flow diagram.

Data were extracted from eligible studies into a standardized data sheet, including information on the first author, publication year, study design, patient characteristics, type of interventions, and outcomes.

### 2.4. Risk of Bias

The risk of bias was assessed independently by two reviewers using the Joanna Briggs Institute’s critical appraisal tools (https://jbi.global/critical-appraisal-tools, accessed 1 May 2024) appropriate for each study type. We used tools for RCTs, quasi-experimental studies, and cohort studies. As the tools do not categorize studies by quality, we established a grading system (low, medium, and high quality). Thirteen aspects were assessed for RCTs. We defined high quality as 12 or 13 fulfilled aspects, medium quality as 10 or 11 fulfilled aspects, and low quality as ≤9 fulfilled aspects. Quasi-experimental studies assessed nine aspects: high quality as 8 or 9 fulfilled aspects and medium quality as 6 or 7 fulfilled aspects. The cohort study assessed eleven aspects.

### 2.5. Analysis

Based on the literature review that we performed before we carried out this systematic review, we assumed that the interventions, the tests to assess the outcomes, the start and intervals of the outcome assessment, and the reporting of the results would be heterogeneous. Therefore, we decided to focus on the outcomes rather than the interventions, as the improvement in outcomes is ultimately the clinically relevant aspect. Based on the assumed heterogeneity, it was not possible to perform meta-analyses or to draw forest plots, and the results are presented descriptively only. Also due to the heterogeneity, we reported the results as significant if it was stated that way in the original publication. If one group performed better that the other, but not statistically significant, we stated it as “trend”. For the readability of the tables, we further did not include the exact result by reporting it as a confidence interval, standard error, or *p*-value, for example. These exact results are available in the Supplementary Material Excel S1. This systematic review is registered on PROSPERO (CRD42024551543). Therefore, no review protocol was prepared.

## 3. Results

**Figure 1:**
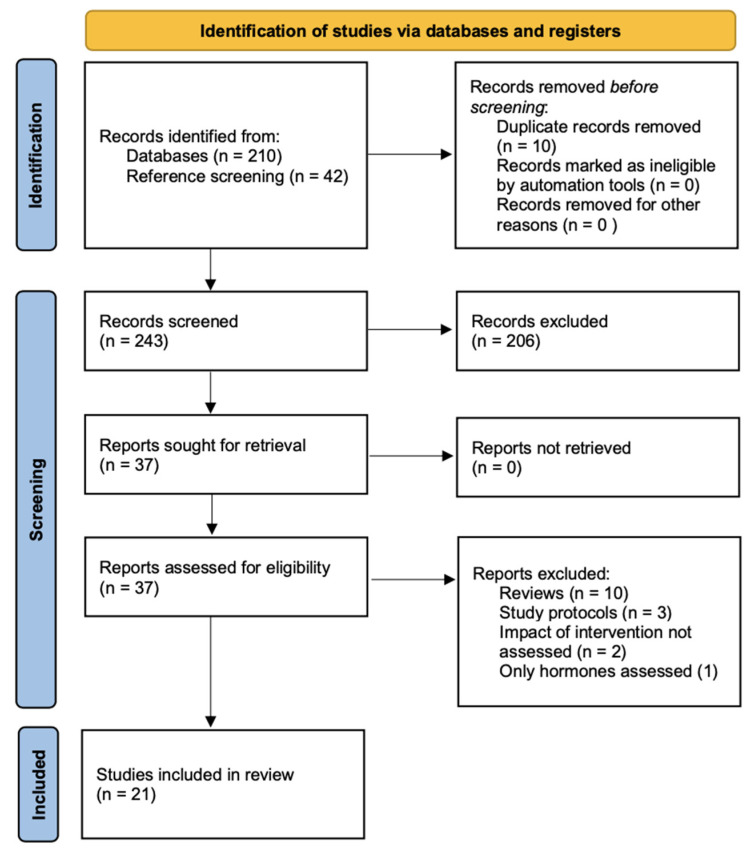
PRISMA flow diagram.

The literature search identified 210 publications. A further 42 publications were added through reference screening, 206 publications were excluded at screening level, and 37 full-text articles were assessed for eligibility. Among these, 21 met the inclusion criteria for this systematic review [15,16,17,18,19,20,21,22,23,24,25,26,27,28,29,30,31,32,33,34,35] (Figure 1).

Half of the included studies were RCTs (10/21), the other half were quasi-experimental studies (10/21), and one was a pre-post cohort study (Table 1). Ten studies examined acute lymphoblastic leukemia patients only. The number of analyzed patients per study ranged from 8 to 170. The quality of the studies included was medium and high in 16 studies (Table 1, Supplemental Table S2). The main aspects resulting in down-grading were that blinding of participants and those who delivered the intervention was not possible or because it was unclear if some aspects were considered in the studies but not reported or were not considered (Supplemental Table S2).

### 3.1. Summary of Interventions

The spectrum of reported physical activity interventions was heterogeneous. We summarized the interventions into five broader categories: (1) physical activity interventions covering aerobic exercise, strength or weight-bearing exercises, and endurance training [15,16,17,18,19,20,21,22,23,24,25], (2) less intensive interventions focusing on stretching, short-burst high-intensity exercises or separate hand or leg function [26,27,28], (3) aerobic exercise only [29], (4) exergaming [30,31,32], (5) yoga [33], and (6) coaching alone [34] (Table 2). Half of the included studies belong to the first category. If mentioned, the duration of the interventions ranged from 3 weeks to 135 weeks (Table 2). Most of the interventions took completely or partially place in the inpatient setting (15/21 studies), and were mostly given as individual sessions and not as group sessions (Table 2). In most studies, the comparators were patients receiving usual care (12/21 studies) or healthy matched controls (5/21 studies), and less frequently the patient itself (e.g., pre- and post-intervention comparisons), or controls receiving general instructions on benefit of physical activity (Table 2). Safety and feasibility were assessed in 15 of the 21 studies and it could be confirmed in all 15 studies with no study reporting accidents or adverse events (Table 2).

### 3.2. Summary of Outcomes

Based on the reported outcomes, we defined the following six outcome categories: (1) cardiopulmonary fitness, (2) muscle strength, (3) physical activity, (4) physical performance, (5) quality of life (QoL) and (6) other outcomes. The outcomes reported in the eligible studies and assigned to each of these categories are summarized in Table 3. The time points of outcome assessments are summarized in Table 2. The spectrum of tests and reported parameters to assess the outcomes was very broad. We therefore only report the most frequent ones in the manuscript, but all tests, outcomes, and effect sizes are reported in the Supplementary Material Excel S1.

#### 3.2.1. Physical Performance

Eleven studies examined a component of physical performance (Table 4). The Timed Up and Go test (TUG) was used most frequently, followed by the Timed Up and Down Stairs test (TUDS), the 6 min walk test (6-MWT), and the Sit to Stand test.

In half of the studies (5/11), patients in the intervention group either significantly improved their physical performance over time or they performed better than controls [18,22,32,33,35]. Additional four studies showed a trend towards improvement in the intervention group over time or compared to controls [17,20,27,35] (Table 4). Thorsteinsson could show a biphasic course with a decline during treatment and an improvement thereafter [23].

#### 3.2.2. Quality of Life (QoL)

Eleven studies examined QoL. Two studies showed no significant difference between intervention and control groups in a cross-sectional comparison of health-related QoL up to 135 weeks of follow-up [15,16]. The same was found for fatigue, behavioral problems, and depressive symptoms.

All eleven studies examined changes in QoL over time—from baseline to different points of follow up (Table 3). Three studies showed an improvement in general QoL in both groups over time [15,17,33], whereas two studies showed no significant difference [27,29]. The results were very heterogeneous in the fatigue subscale. One study reported an increase in fatigue scores in both groups [15], one reported no change in both groups [30] and three an improvement in the intervention group [25,32,34] (Table 5).

#### 3.2.3. Physical Activity

Ten studies assessed physical activity. The pattern of change in physical activity between the intervention and control group and within each group over time were very heterogeneous (Table 6).

Only the results by Kowaluk et al. showed significant differences between the intervention and control groups in the short-term, where patients from the intervention group were more physically active, reported in the HBSC questionnaire [31]. Contrary, three studies showed no significant differences in the short- or long-term assessment between both groups [15,30,31], including Kowaluk et al. with no significant difference in the long-term [31]. Looking at changes in physical activity over time, only Fiuza-Luce and Masoud et al. reported a significant increase over time in the intervention group [17,32], with two additional studies reporting a trend towards an increase in the intervention group [16,35]. The interventions in these four studies included circuits including aerobic and weight-bearing exercises, video games, and an exercise program not further specified (Table 2). Two studies reported a larger increase in the control group [17,30].

#### 3.2.4. Muscle Strength

Nine studies examined muscle strength, looking into different muscle groups and thus using different tests to assess the effect of the interventions (Table 7). Grip strength, knee extension, and ankle dorsiflexion were examined in three studies each. Four studies assessed combinations of multiple tests over time (e.g., upper and lower body muscle strength, 5-repetition maximum). No assessment was used twice. Muscle strength significantly increased in five studies, from baseline to last follow-up. The underlying interventions in these five studies were aerobic and weight-bearing circuits [15,17,21,22] and a combination of stretching, strengthening, and aerobic fitness [27] (Table 2).

#### 3.2.5. Cardiopulmonary Fitness

Six studies examined cardiopulmonary fitness and performed cardiopulmonary exercise tests (CPET). Peak oxygen uptake (VO_2_peak) was the only parameter assessed in all six studies. No study could show a significant difference between the intervention and control group. The same was true for differences within each group over time. However, four studies showed a trend towards improvement in VO_2_peak in the intervention group (Table 8). The interventions in these studies were aerobic and weight-bearing/muscle strength circuits with or without balance exercise [17,18,20] and interactive videogaming [31] (Table 2).

#### 3.2.6. Other Outcomes

Four studies assessed flexibility, three assessed motor performance and one assessed acute toxicities using the Common Terminology Criteria for Adverse Events (CTCAE).

Among other parameters, flexibility was assessed in all studies using active or passive ankle dorsiflexion. Dorsiflexion decreased in two studies over time [16,26] remained stable and increased in one study each [27,33]. The decrease could be measured in the intervention and control group in both studies, with the second assessment at 135 weeks and 2 years, respectively (Table 9). The examined population were leukemia patients in both studies.

Motor performance was assessed by four different test batteries. None of the three studies reported a significant difference in motor performance between the intervention and control group. Hartman et al. reported a trend towards improvement over time in the overall cohort, but no difference between both groups [26]. Contrary, the performance decreased over time in both groups combined in the study by Hamari et al.; again, with no difference between both groups (Table 9) [30]. Both studies had a follow-up of at least one year. The interventions differed with exergaming in one and exercise program, including stretching and short-burst high-intensity elements in the other study (Table 2).

Munise et al. reported their results on acute toxicities descriptively only [19]. The 8-week individualized program consisted of aerobic, resistance, and flexibility exercise and resulted in a reduction in severe fatigue in the intervention group (Table 5). The outcome was assessed after 10 weeks (Table 2).

## 4. Discussion

In this systematic review of studies on physical activity interventions during cancer treatment in children and adolescents, we identified 21 publications. We could identify six different categories of interventions: (1) physical activity sessions covering aerobic exercise, strength or weight-bearing exercises, and endurance, (2) less intensive physical activity sessions focusing on stretching, short-burst high-intensity exercises or separate hand or leg function, (3) aerobic exercise only, (4) exergaming, (5) yoga, and (6) coaching alone. We further identified six categories how the impact of physical activity can be assessed: (1) cardiopulmonary fitness, (2) muscle strengths, (3) physical activity level, (4) physical performance, (5) quality of life, and (6) other outcomes. These categories highlight the broad and heterogeneous spectrum used today. The clinically important aspects regarding physical activity interventions in this patient population include feasibility, adverse events, and clinical impact.

### 4.1. Feasibility of Physical Activity Interventions

Results from this systematic review show that physical activity interventions for children and adolescents undergoing cancer treatment are both safe and feasible. In support of this, 15 out of 21 studies confirmed the safety and feasibility of engaging in physical activity during cancer treatment.

Another systematic review by Grimshaw et al. (2016) came to the same conclusion by analyzing 11 quantitative and 1 qualitative study, concluding that such interventions were acceptable to both children and their parents, and could be successfully implemented in hospital settings [36]. Another example that physical activity during treatment is both feasible and beneficial is the ActiveOncoKids network in Germany. They aim to provide exercise opportunities for pediatric oncology patients throughout their treatment journey [37]. A significant achievement of ActiveOncoKids is the development of consensus-based guidelines for implementing movement and exercise interventions in pediatric oncology. These guidelines offer eleven recommendations addressing the importance of exercise during treatment, program design, safety considerations, and strategies to overcome barriers to participation. They emphasize that exercise interventions should be tailored to individual patient needs, considering factors such as physical and mental impairments, inactivity levels, and clinical restrictions [38].

Despite the promising evidence, several barriers to implementing physical activity interventions remain [39]. Individual factors such as the child’s physical condition, treatment side effects, psychological state, and type of physical activity can influence participation. Environmental or family factors can have an impact too. Logistical challenges may include scheduling of the sessions in general and being flexible if the actual health status does not allow physical activity or the activity needs to be adapted at short notice. Lastly, institutional barriers may exist, such as the availability of resources (space and manpower) and recognizing the necessity of such interventions regarding the economic thinking and the fear of too high costs and expenses. Addressing these barriers requires a tailored approach that considers individual patient needs and local circumstances.

### 4.2. Feasibility of Outcome Assessment

Studies have demonstrated that assessing physical fitness, functional capacity, and QoL in this population is feasible; however, logistical and methodological barriers remain. Factors influencing the feasibility of outcome assessment and the participants’ ability to engage in assessments at consistent and predefined time points include: (1) the heterogeneity of treatment protocols with different intensity of the treatment administered and different intervals between phases of intensive treatment and phases of recovery, (2) the disease severity, and (3) the age of the child or adolescent. Various assessment tools, such as cardiopulmonary exercise testing, six-minute walk tests, and different strength measurements, have been successfully implemented in research settings. Other assessments proved to be less feasible. For example, Thorsteinsson et al. excluded the modified Andersen test from their test battery, as they deemed the test not compatible in children with cancer and it was not possible to rate the fitness as with VO_2_peak [23]. Despite these challenges, research has shown that outcome assessments can be integrated into clinical and rehabilitation programs with appropriate modifications. For instance, the study by Caru et al. demonstrated the feasibility of a multidisciplinary physical activity program in pediatric oncology, reporting an adherence rate of about 40% despite hospital-specific restrictions and organizational conflicts [40]. While assessing outcomes in physical activity interventions for children and adolescents with cancer during therapy is feasible, it requires careful consideration of methodological and logistical challenges. Addressing these challenges through innovative approaches and standardized protocols will enhance the quality and applicability of future research in this field.

### 4.3. Adverse Events

The findings of our systematic review underscore the feasibility of integrating physical activity programs into oncology care without posing additional risks to patients. This is consistent with results from other studies that have shown that physical activity in children and adolescents with cancer undergoing treatment is considered safe [7,41]. The absence of reported injuries or negative effects suggests that these interventions can be safely implemented when appropriately designed and monitored. A key factor contributing to the safety of these programs is their individualized approach [6]. In all studies, interventions have been carefully adapted to patients’ age, physical capability, and clinical status, ensuring that activities are neither excessively demanding nor detrimental to their health. This personalized approach accounts for the varying degrees of treatment-related fatigue, immunosuppression, and other side effects that may impact a child’s ability to participate. Some studies even took the thrombocyte count into account and reduced or omitted intense sessions when the counts were too low [42]. Additionally, supervised sessions led by trained professionals further enhance safety by allowing for real-time adjustments based on each patient’s condition [6]. While the current evidence supports the safety of physical activity during cancer treatment, this topic was not mentioned in all studies included in this systematic review, and future studies should continue to monitor potential risks systematically. Standardized safety reporting across studies will be essential to further validate these findings and optimize intervention protocols.

### 4.4. Clinical Impact of Physical Activity Interventions

Based on the results of our systematic review, physical activity interventions have demonstrated physical and psychological benefits for children and adolescents undergoing cancer treatment. Engaging in structured exercise programs has been associated with improvements in physical fitness, muscle strength, and functional mobility, which are often compromised due to the effects of chemotherapy, radiation, and prolonged hospital stays [7]. This systematic review showed the largest amount of improvement in cardiopulmonary fitness, physical performance, and muscle strength.

While many studies report beneficial effects, it is important to note that improvements are not always significantly larger in intervention compared to the control group. Some studies, including some of this review, observed positive changes in both groups. For example, Braam et al. showed an increase in cardiopulmonary fitness in both groups [15]. Possible reasons for this observation could be that the initial shock of the diagnosis and feeling insecure in the new situation contribute to a decline in physical activity in general. Following this initial phase and when patients become more familiar with the new situation, they automatically become more active again. This might explain the improvement not only in the intervention, but also in the control group. The observation that improvements in the intervention group are not always significantly better than in controls could be explained by the rather small samples examined. In addition, children are generally less frail than adults and might cope better with phases of reduced activity or inactivity. Natural maturation and aging may also be another possible cause of improvement over time for both groups.

The same as for physical fitness could also be shown for quality of life, where three studies have shown that there was an improvement in the intervention and control group [15,17,33]. Again, this may be due to natural recovery processes, variations in usual care, or the influence of other supportive therapies. Additionally, the clinical impact of physical activity interventions remains limited by small study populations and variations in study protocols, making it difficult to draw definitive conclusions.

### 4.5. Aspects for the Future

While existing research supports the feasibility, safety, and potential benefits of physical activity interventions in children and adolescents with cancer, several important aspects remain to be addressed in future studies. One key area is the need for larger, multicenter randomized controlled trials to strengthen the evidence. Many current studies are limited by small sample sizes, short intervention durations, and heterogeneous study designs, making it difficult to draw definitive conclusions about the long-term clinical impact of physical activity. Understanding the optimal type, intensity, and duration of physical activity interventions will be key to maximizing their impact on clinical outcomes. Future research should focus on standardized protocols to ensure comparability and reproducibility of findings across different patient populations and treatment settings. Furthermore, future research should investigate personalized approaches to exercise prescription. Given the variability in cancer types, treatment regimens, and individual patient conditions, tailoring physical activity interventions based on specific needs and treatment phases may optimize their effectiveness. Studies should explore how different intensities, frequencies, and modalities of exercise impact various patient subgroups to develop more individualized recommendations. Lastly, the current evidence allows no conclusion on whether the interventions have a long-lasting impact on physical activity adherence or whether they have an impact on physical activity levels once the intervention is terminated. The duration of the intervention in the reported studies was very broad and ranged from 3 to 135 weeks. In terms of the impact of the interventions, the outcomes were assessed in most studies during or shortly after the end of the intervention. The longest follow-up was two years after the intervention in one study. Therefore, future studies should also investigate the aspect of long-lasting effects and whether the level of physical activity might also have an impact on other social aspects (e.g., employment),

### 4.6. Strength and Limitations

A significant strength of this review is its robust methodological approach. The screening and data extraction process was conducted by two independent reviewers, ensuring thorough and unbiased selection of studies. In cases of disagreement, a third independent reviewer was involved to make the final decision, enhancing the reliability of the study selection process. This systematic and transparent approach minimized the risk of selection bias and improved the overall quality of the review. Furthermore, a detailed quality assessment of the included studies was performed, evaluating the internal and external validity of the studies. This process helped ensure that the studies included in the review were both reliable and relevant to the research question. The quality assessment allowed for a critical evaluation of the studies’ methodologies and their ability to provide meaningful insights into the clinical impact and safety of physical activity interventions in pediatric oncology.

Despite these strengths, several limitations must be acknowledged. Searching PubMed as the only database could be considered a limitation. However, reference secreening of identified (systematic) reviews and guidelines/recommendations was performed. One major limitation is the large variety of interventions and outcome measures used across the studies. This diversity made it difficult to draw clear and definitive conclusions regarding the effectiveness of physical activity interventions. The lack of consistency in outcome measures prevented the possibility of conducting a meta-analysis, which could have provided a more comprehensive summary of the evidence. The variation in interventions, including different exercise regimens, intensities, and duration, and outcome measures also complicated direct comparisons between studies. As a result, it was not possible to determine the superiority or inferiority of specific physical activity interventions. For the same reason, it was also not possible to examine differences bewteen different types of cancer. Additionally, many studies included small sample sizes and heterogeneous patient populations, which limited the generalizability of the results. The lack of statistical power often led to the reporting of only trends or small effects, making it difficult to draw firm conclusions. Some studies even reported results descriptively without statistical analyses, further limiting the ability to evaluate the true impact of physical activity interventions on health outcomes in this population.

## 5. Conclusions

In conclusion, physical activity interventions for children and adolescents undergoing cancer treatment have been shown to be both safe and feasible, but few studies were shown to be significantly effective. While most studies report improvements in the intervention groups, similar effects were also observed in control groups. A key limitation remains the small sample sizes in existing studies, which restrict the generalizability of findings. These limitations highlight the need to perform controlled studies, as it will be impossible to estimate the effect of the intervention witout assessing the natural change. Despite these challenges, physical activity can be endorsed as a beneficial component of pediatric oncology care. Further research is essential to assess the long-term impact and sustainability of these interventions to optimize their effectiveness and implementation in clinical practice. Future studies should focus on identifying and mitigating barriers, tailoring interventions to individual needs, and integrating behavior change techniques to promote sustained engagement in physical activity during and after cancer treatment.

## Figures and Tables

**Table 1:** Main characteristics of the 21 included studies.

Author, Publication Year, Country	Study Design Year of Study Conduct	Analyzed Cohort (Intervention, Control) Diagnosis	Age at Intervention (Years)	Quality
Braam, 2018, The Netherlands [15]	Prospective multi center RCT, 03/2009-07/2013	68 (30, 39), different cancer types	mean: 13.2 range: 8–18	medium
Cox, 2018, Canada, USA [16]	Prospective multi center RCT, 2.5 years (years not reported)	77 with all assessments completed (36, 41), ALL	range: 4–18.99	medium
Fiuza-Luce, 2016, Spain [17]	Prospective single center RCT, 09/2012–09/2015	49 (24, 25), extracranial solid tumors	range: 4–18	medium
Fridh, 2023, Denmark [18]	Prospective multi center non-RCT, 01/2013–02/2018	108 (75, 33) and 64 ambassadors, different cancer types	intervention: 13.4 ± 3.1 control: 13.5 ± 2.5	medium
Hamari, 2019, Finland [30]	Prospective RCT, years unkown	35 (17, 18), ALL and non-CNS cancers	intervention: mean 7.8 (3–16) control: mean 7.9 (3–15)	medium
Hartman, 2009, The Netherlands [26]	Prospective single center RCT, 04/2001–09/2004	51 (25, 26), ALL	intervention: 5.3 (1.3–15.6) control: 6.2 (1.7–17.1)	medium
Hook, 2019, USA [34]	Prospective multi center quasi-experimental, years unkown	57 (30, 27), any cancer except for CNS, bone extremity tumors, or cancers with bone extremity metastasis	intervention: 12.2 control: 12.8	medium
Khodashenas, 2017, Iran [29]	Prospective single center non-RCT, 2015–2016	20 (10, 10), ALL	control: 8.8 (5–12) intervention: 10.1 (5–12)	medium
Kowaluk, 2022, Poland [31]	Prospective single center RCT, 01/2019–01/2020	21, leukemia	intervention: 11.3 ± 1.9 control: 10.08 ± 1.9	low
Marchese, 2004, USA [27]	Prospective single center RCT, years unkown	28 (13, 15), ALL (maintenance)	mean: 7.7 (4–18)	low
Masoud, 2023, Saudi Arabia [32]	Prospective single center RCT, 11/2019–11/2020	45 (22 group 1, 23 group 2), ALL	mean: 9 (±2.35) range: 6–14	low
Moyer-Mileur, 2009, USA [35]	Prospective single center RCT, years unkown	13 (6, 7), ALL (maintenance)	range: 4–10	low
Munise, 2024, Australia [19]	Prospective single center RCT, 2018–2021	39, Hodgkin and Burkitt lymphoma, sarcoma, CNS tumor, germ cell tumor, leukemia, melanoma	range: 15–25 intervention: 21.9 (3) control: 20.3 (2.7)	medium
Nielsen, 2020, Denmark [20]	Prospective multi-center non-RCT, 2013–2018	170 (120, 50), Any cancer diagnosis, LCH, MDS; treated with chemo- and/or radiotherapy	range: 6–18	high
Perondi, 2012, Brazil [21]	Prospective single center quasi-experimental, years unknown	6, ALL (maintenance)	range: 5–18	high
San Juan, 2007, Spain [22]	Prospective single center quasi-experimental, years unknown	7, ALL (maintenance)	range: 4–7	medium
Thorsteinsson, 2017, Denmark [23]	Prospective single center controlled and mixed methods intervention 01/2013–04/2016	75, any cancer diagnosis, LCH, MDS; treated with chemotherapy	median: 11 yr range: 6–18	high
Vriens, 2022, Belgium [28]	Prospective single center cohort study, 01/2013–12/2018	62, ALL and lymphoblastic lymphoma	mean: 7.6 ± 4.3 range: 2.1–18	low
Winter, 2013, Germany [24]	Prospective single center non-randomized comparative cohort study, 07/2006–12/2009	31 (16, 15), malignant bone tumor in the lower extremity	intervention: 13.5 control: 14.0	high
Wurz, 2014, Canada [33]	Prospective feasibility study, years unkown	8, different types of cancer	mean: 11.88	high
Yeh, 2011, Taiwan [25]	Prospective quasi experimental feasibility study, 06/2006–07/200	22 (12, 10), ALL (maintenance)	control: 12.48 intervention: 11.01	high

Abbreviations: ALL, acute lymphoblastic leukemia; CNS, central nervous system; LCH, Langerhanscell histiocytosis; MDS, myelodysplastic syndrome; RCT, randomized controlled trial.

**Table 2:** Overview of the interventions, interval from diagnosis to intervention, and time of outcome assessment in the 21 included studies.

Author	Description of the Intervention; Safety and Feasibility of the Intervention	Place of Intervention, Setup, Comparator, Duration of Intervention	Intervention Frequency	Interval from Diagnosis to Intervention	Time of Assessment	Outcome Category
Braam [15]	Individualized aerobic and weight-bearing (circuit) exercise training; Feasible	Inpatient and at home, alone, usual care, 12 weeks	Inpatient: 2 × 45 min/week At home: 3 × 11 min/week	Under treatment or within one year post-treatment	Baseline, 4 and 12 months	Cardiopulmonary fitness, muscle strength, physical activity, QoL
Cox [16]	Physical therapy exercises: strength, range of motion, gross motor skills, endurance; Not mentioned	Alone, usual care, 135 weeks	At home: 5 × 30 min/week Physiotherapist: weekly weeks 1–4; 1× every 2 weeks for week 5–8, monthly weeks 9–135	Within 10 days from start of chemotherapy	1–2 days prior to the baseline, 6–7 weeks after chemo start, 8–9 weeks after completion of induction therapy, ~135 weeks after chemo start	Muscle strength, physical activity, physical performance, QoL, other outcomes
Fiuza-Luces [17]	Aerobic and muscle strength: each session with (1) approx. 30 min aerobic exercise (cycle-ergometer, treadmill running, arm cranking, aerobic games); (2) approx. 30 min strength (shoulder, chest and leg presses, rowing, knee extension and flexion, and abdominal, lumbar and shoulder adduction); Safe	Inpatient, alone and group, usual care, 19 ± 2 week	3 × 60–70 min/week	Not mentioned	Baseline, post-treatment and detraining	Cardiopulmonary fitness, muscle strength, physical activity, physical performance, QoL
Fridh [18]	Individualized physical activity program (RESPECT activity program); Safe and feasible	Inpatient, alone, usual care and ambassadors, duration not mentioned	Alone: 3 × 5–30 min/week Group: 2 × 30–120 min/week	Not mentioned	1-year post treatment ± 10 days	Cardiopulmonary fitness, muscle strength, physical performance
Hamari [30]	Nintendo WiiTM games; Feasible	Inpatient and at home, alone, written advice for PA of 30 min/day, entire treatment period	30 min/day, daily	Mean (SD) of 15.4 (13.3) days from initial diagnosis	First week of intervention and after one year	Physical activity, QoL, other outcomes
Hartman [26]	Exercise program for hand and leg function; stretching for ankle dorsiflexion; short-burst high-intensity exercises; Not mentioned	At home, usual care 2-year treatment period	Exercises for hand and leg function: 1×/day Stretching and jumping: 2×/day	Not mentioned	At diagnosis, 32 weeks after diagnosis, 1 year after diagnosis, on cessation of therapy (2 years after diagnosis) and 1 year after cessation of therapy	Other outcomes
Hooke [34]	Coaching on physical activity and fatigue (motivational interviewing); Safe	Inpatient, usual care, duration not mentioned	Not mentioned	Second month of cancer treatment	2nd, 4th, and 6th months of cancer treatment	Physical activity, QoL
Khdashenas [29]	Aerobic exercise: walking, running, different forms of playing; Not mentioned	NA, usual care, 12 weeks	3 × 60 min/week	Not mentioned	Before and after intervention/training program	QoL
Kowaluk [31]	Interactive video games: beach volleyball, tennis, river rush, reflex ridge; Safe and feasible	Inpatient, no video game kit, 4 weeks	3×/week	Within 6 months from diagnosis	CPET at baseline and 14 months	Cardiopulmonary fitness, physical activity
Marchese [27]	Stretching and strengthening: 5 days/week bilateral ankle dorsiflexion stretching; 3 days/week bilateral lower extremity strengthening; daily aerobic fitness (e.g., walking, biking, or swimming); Not mentioned	Inpatient and at home, alone, usual care, 4 months	20–60 min	Second half of maintenance therapy	Immediately after the initial testing, and 2, 4, 8, and 12 weeks later	Muscle strength, physical performance, QoL, other outcomes
Masoud [32]	Exergaming of moderate intensity; Not mentioned	Inpatient, alone, one PA instruction and advice to practice 60 min twice a week, 3 weeks	60 min per session, 2×/week	Not mentioned	First, third, and fifth weeks of intervention	Physical activity, physical performance, QoL
Moyer-Mileur [35]	Exercise and nutrition program; Feasible	At home, alone, standard dietary recommendations, multivitamin and PA as tolerated, 12 months	15–20 min per session, 3×/week	Beginning maintenance therapy	At enrollment (baseline) and at 3, 6, 9, and 12 months thereafter.	Physical activity, physical performance
Munise [19]	Individual program: aerobic, resistance and flexibility; Feasible	Inpatient, alone, usual care, 10 weeks	2×/week	Not mentioned	Weekly over 10 weeks	Other outcomes
Nielsen [20]	Individualized program: cardiorespiratory fitness, muscle strength and balance; Safe and feasible	Inpatient, alone and group, usual care and ambassadors, duration not mentioned	Alone: 3 × 30–120 min/week Group: 2 × 30–120 min/week	From diagnosis to 6 months after diagnosis	Baseline within 31 days of diagnosis, 3 months ± 30 days after diagnosis, 6 months ± 30 days after diagnosis	Cardiopulmonary fitness, muscle strength, physical performance
Perondi [21]	High-strength exercises and moderate aerobic exercise: 10 min treadmill warm-up, 30 min resistance training (e.g., leg press), 20 min treadmill aerobic training, 5 min stretching; Safe	Inpatient, alone, pre-versus post-intervention, 12 weeks	2 × 60 min/week	30 to 116 weeks of treatment	At baseline and after 12 weeks (after intervention)	Muscle strength, QoL
San Juan [22]	Start and end of sessions: low-intensity 15 min warm-up and cool-down (cycle-ergometer, stretching). Strength: 11 exercises for major muscle groups Resistance individualized. Stretching: during the rest periods between exercises. Aerobic excercise: cycle-ergometer, running, walking, aerobic games; Safe	Inpatient, group, pre- versus post-intervention, 8 weeks	3 × 90–120 min/week	18 and 24 months after start of treatment	Pretraining and 8 weeks later (post training)	Muscle strength, physical performance
Thorsteinsson [23]	Muscle strength, cardiorespiratory fitness and balance; Safe and feasible	Inpatient, alone, healthy classmates, age- and sex-matched population, duration not mentioned	Alone: daily Group: 2×/week	Not mentioned	At diagnosis, 3 and 6 months after diagnosis, 1 year after cessation of treatment	Cardiopulmonary fitness, physical performance
Vriens [28]	Physiotherapy during intensive treatment; Not mentioned	Inpatient, alone, healthy peers, duration not mentioned	2–5 times/week	1 to 130 weeks after diagnosis	During treatment: week 1, 5, 11, 22, 29, 52, and week 78 After treatment: week 104, week 130	Muscle strength, physical performance
Winter [24]	Adolescents: structured individualized program with warm up, strength, endurance, and stretching. Younger children: activate by games; Feasible	Inpatient, alone, usual care, duration not mentioned	30–60 min; 1×/day	Not mentioned	During treatment: 6 weeks, 3 months, and 6 months post-surgery. After end of treatment: 12 and 18 months follow-up	Physical activity
Wurz [33]	Yoga sessions with warm-up, different poses, group activity, final resting pose; Safe and feasible	Local community, alone and group, pre- versus post-intervention, 12 weeks	2 × 60 min/week	Not mentioned	At baseline and post-intervention (within 2 weeks of completing the 12-week intervention)	Physical activity, physical performance, QoL, other outcomes
Yeh [25]	Exercise video with individualized instructions: warm-up (5 min), aerobic exercise (25 min), cooldown (5 min); Safe and feasible	At home, alone, usual care, matched for age and sex, 6 weeks	3 × 30 min/week	Not mentioned	Baseline, weekly for week 1–5, post-test (6 weeks), and 1-month follow-up	QoL

Abbreviations: PA, physical activity.

**Table 3:** Overview of the six outcome categories.

Category	Outcomes
(1) Cardiopulmonary fitness	VO_2_peak Heart rate VO_2_, VCO_2_, VE, VE/VCO_2_, MET
(2) Muscle strength	Hand-held dynamometer 10/6-repetition maximum (10/6-RM) Push ups (Moyer-Mileur)
(3) Physical activity	Step count (accelerometer) Godin-Shepard leisure time physical activity questionnaire (GSLTPAQ) HBSC questionnaire ACTIVITYGRAM questionnaire
(4) Physical performance	Six-minute-walk test Sit-to-stand test Timed-up-and-go test (TUG) timed up and down stairs test (TUDS) Andersen Fitness test Standing broad jump (SBJ) Back-Saver Sit and Reach Test Progressive Aerobic Cardiovascular Endurance Run
(5) Quality of life	PedsQL generic score Child Health Questionnaire Fatigue scale of PedsQL (QLMFS) Childhood/Adolescence Fatigue Scale Common Terminology Criteria for Adverse Events (CTCAE) Youth Self-Report Children’s Depressive Inventory Children’s Effort Rating Table Scale (CERT)
(6) Other outcomes	
Motor performance	Bruininks-Oseretsky Test of Motor Proficiency Short Form (BOTSF-2) Bayley Scales of Infant Development (BSID-II) Children > 4 years: Movement assessment Battery for Children
Balance and coordination	Flamingo balance
Flexibility	Hip, knee and ankle-dorsiflexion scores Knee extension Ankle range of motion Sit and reach (Moyer-Mileur)

**Table 4:** Results of included studies reporting on physical performance.

Author	How Outcome Assesseed	Results Overview
Cox [16]	6-MWT	No significant difference between intervention and control group in the 6-MWT; trend towards longer distance in control group.
Fiuza-Luces [17]	3 m TUG and TUDS	No significant group–time interaction effect for 3 m TUG and TUDS; trend towards longer performance time in intervention group.
Fridh [18]	Sit-to-Stand-Test 3 m TUG	Significantly higher Sit-to-Stand score in intervention compared to control group. Significantly faster 3 m TUG in intervention compared to control group.
Marchese [27]	TUDS and 9 min run-walk test	No significant differences between groups for TUDS and 9 min run-walk test from baseline to 4 months. Trend towards larger decrease (delta) in time used for TUDS and larger increase in 9 min run-walk distance in intervention group.
Masoud [32]	6-MWT	No significant differences between intervention and control group in baseline 6-MWT. Significant effects of time and time x intervention interaction on 6-MWT. Intervention group with significant improvement in endurance (longer distance in 6-MWT) from T1 to T2, T1 to T3, and T2 to T3.
Moyer-Mileur [35]	Progressive Aerobic Cardiovascular Endurance Run (PACER)	Significant greater PACER laps at 12 months in intervention compared to control. For 6 to 12 months, trend towards larger increase in PACER laps in the intervention compared to control group. For 0 to 6 months and 0 to 12 months, trend towards larger increase in control compared to intervention group.
Nielsen [20]	TUG and Sit-to-Stand-Test	No difference in changes over time in TUG and sit-to-stand between intervention and control group. Intervention group performed better at all three time points in both tests. At baseline, intervention and control group performed significantly worse than healthy age- and sex-matched children in both tests.
San Juan [22]	3-and 10 m TUG test TUDS test	Significant improvement in 3- and 10 m TUG test from pre- to post-training. Trend towards improvement in TUDS from pre- to post-training.
Thorsteinsson [23]	TUG Sit-to-Stand-Test Andersen Fitness test	Decline in both tests during treatment (from diagnosis to 3 and 6 months); improvement thereafter (descriptive only). Modified Andersen test excluded from test battery, as not compatible in children with cancer and not possible to produce a fitness rating like VO_2_peak test.
Vriens [28]	Standing broad jump (SBJ) 6-MWT	Significantly lower 6-MWT at all weeks in patients compared to healthy peers. Decrease in 6-MWT after induction treatment, but no significant change overall. Significantly lower SBJ scores in patients compared to healthy peers at all measured timepoints, except at week 130. Strongest decrease in SBJ scores after induction, smaller decrease after reinduction.
Wurz [33]	3 min TUG	Significant improvements in TUG test from baseline to post-intervention.

**Table 5:** Results of included studies reporting on quality of life.

Author	How Outcome Assesseed	Results Overview
Braam [15]	General HRQOL: Dutch version of PedsQL (Generic Core Scales). Fatigue: Overall-fatigue score by child self-report PedsQL. Behavioral problems: Youth Self-Report (athletic competence, global self-worth, total problems). Depressive symptoms: Children’s Depressive Inventory	No significant differences at 4 and 12 months between intervention and control group for HRQOL, fatigue, behavioral problems and depressive symptoms. In intervention and control group, general HRQOL significantly increased over time. Trend towards increase in fatigue scores in both groups over time. Significant decrease in depressive symptoms in control group and trend towards decrease in intervention group. Trend towards decrease in total behavioral problems in both groups over time.
Cox [16]	General HRQOL: Child Health Questionnaire (parent and child form)	Children consistently scored their QoL better than parents at all time points (physical functioning, emotional–behavioral role, physical role, and bodily pain). No significant difference in all HRQOL subscales between children from the intervention and control group.
Fiuza-Luces [17]	QOL: Spanish version of PedsQL, Cancer Module 3.0 (parent and child form)	Training-induced improvement in patient-reported QoL, but no significant group–time interaction effect for patient- or parent-reported QoL.
Hamari [30]	Fatigue: PedsQL Multidimensional Fatigue Scale	Change in fatigue scores between pre- and post-intervention did not differ between both groups.
Hooke ° [34]	Fatigue: Childhood Fatigue Scale (6 to 12 years); Fatigue Scale-Adolescent (13 to 18 years)	Trend towards decrease in fatigue T scores during first 6 months of treatment for all diagnoses combined. No significant difference in fatigue T scores at 4 months between intervention group and historical control. No relationship between change in physical activity and fatigue T score (e.g., physical activity increase results in fatigue decrease).
Khodashenas [29]	QoL: PedsQL version 4 (physical, emotional, social, and school functioning)	No significant difference between QoL index in the intervention and control group between pre- and post-test.
Marchese [27]	QoL: PedsQL version 3.0	No significant differences between intervention and control group for child cancer PedsQL, child general PedsQL, parent cancer PedsQL, and parent general PedsQL from pre- to post-test.
Masoud [32]	Fatigue: Pediatric quality of life multidimensional fatigue scale (peds QLMFS) including general, sleep/rest, cognitive and total fatigue	No significant differences between intervention and control group in fatigue at baseline. Significant effect of exergaming on general and total fatigue. Significant effects of time on total and sleep/rest fatigue. The effect of time×intervention interaction was significant for all dimensions of fatigue. Intervention group with significant decrease in all fatigue subscales compared to controls after 4 weeks.
Perondi [21]	QoL: Brazilian PedsQL (generic, fatigue and cancer), patient- and parent-reported	Trend towards better scores in all three domains and reported by patients and parents. Only significant improvement in the fatigue subscale, reported by parents.
Wurz [33]	HRQOL: PedsQL 4.0 General Module, patient- and parent-reported	Significant patient-reported improvement in total HRQL and on psychosocial HRQL. Trend towards improvement in all other domains. Significant parent-reported improvements in child’s total HRQL, psychosocial, physical, and school HRQL. Trend towards improvement in the other modules.
Yeh [25]	Fatigue: PedsQL Multidimensional Fatigue Scale (general, sleep/rest and cognitive fatigue)	Significantly fewer general fatigue symptoms in intervention group compared to control group at last follow-up. Decrease in general fatigue in intervention group and increase in control group at last follow-up (descriptive only). Initial decrease in sleep/rest fatigue in both groups, further decrease in intervention and flattened in control group (descriptive only). Decrease in cognitive fatigue in intervention group, but initial decrease followed by increase in control group (descriptive only). Mean scores of all three fatigue subscales decreased from post-test to 1-month follow-up in intervention group, which did not appear in control group.

° results shown for all cancer diagnoses combined; separate results for leukemia, lymphoma and solid tumor patients in Supplementary Material.

**Table 6:** Results of included studies reporting on physical activity.

Author	How Outcome Assesseed	Main Results *
Braam [15]	Actigraph accelerometer: waist belt, worn during daytime on 4 consecutive days; expressed as mean counts per minute (CPM): physical activity score including horizontal, vertical and depth motion scores	At 4 and 12 months, no significant differences between intervention and control group in mean CPM. Significant increase in mean CPM in control group from baseline to 12 months; number of children wearing actigraph decreased over time. Trend towards increase in mean CPM in intervention group from baseline to 12 months.
Cox [16]	Actigraph accelerometer: worn for 7 days; algorithms provided estimated energy expenditure (EE) data for each minute, summary data including total daily EE estimates and percentage of time spent in different intensities of activity (rest, light, moderate, vigorous).	Trend towards increase in activity level in intervention and control group from baseline to end of therapy. Activity levels declined in both groups at T2.
Fiuza-Luces [17]	Actigraph accelerometer: worn for up to 7 consecutive days, minimum of 4 days; expressed as average and count ranges for different activity intensities (light, moderate, vigorous physical activity)	No significant difference in time spent inactive between intervention and control group; also not over time. No significant difference in time spent with moderate-vigorous physical activity between intervention and control group; significant improvement in both groups over time.
Hamari [30]	Actigraph accelerometer: waist belt when being awake during first week of the intervention; reported as mean activity counts/h. Activity diary: filled out in 10 min periods during first week of intervention; reported as mean time/day physically active. Metabolic equivalent (MET) questionnaire: leisure-time physical activity in MET h/week; three questions about physical activity intensity, duration, and frequency	No significant difference in median accelerometer counts between intervention and control group during first week of intervention. Control group with significantly higher median accelerometer counts at one year. Trend towards larger increase in median accelerometer counts over time in control group. No significant difference in self-reported physical activity (diary) between intervention and control group. Change in MET h/week did not differ between both groups
Hooke ° [34]	Actigraph accelerometer: waist belt, worn on 3 consecutive days Modified Leisure Score Index of the GLTEQ	No significant changes in Leisure Score Index over 6 months in intervention group. Trend towards higher self-reported scores in intervention group at 4 months compared to historical controls. Numbers of steps per day did not change significantly over time.
Kowaluk ^‡^ [31]	Health Behaviour in School-Aged Children Questionnaire (HBSCQ): number of days per week where child performed physical activity (MVPA) for at least 60 min (HBSC 1); frequency of undertaking vigorous physical activity (HBSC 2)	Immediately after intervention, intervention group was significantly more physically active than controls. After 14 months, no significant difference between intervention and control group, but trend towards higher physical activity in intervention group. Intervention group was significantly more physically active immediately after, compared to pre-intervention. Physical activity in intervention group at 14 months comparable to level immediately after end of intervention. Physical activity level did not increase in control group in short term, but, significantly at 14 months.
Masoud [32]	GSLTPAQ to estimate the Leisure Time Score and categorization into active, sufficiently active, and insufficiently active	Non-significant differences between intervention and control group for physical activity level at baseline. Significant increase in physical activity in intervention group compared to control over time. No significant improvement in physical activity from insufficiently active to active or sufficiently active.
Moyer-Mileur [35]	ACTIVITYGRAM questionnaire: food intake and three-day activity (type of activity, intensity (light, moderate, or vigorous), and duration in minutes starting from the time the child awoke until bedtime). Only moderate-to-vigorous activities included in the analysis. Pedometer	Self-reported activity minutes significantly greater in intervention group at 12 months compared to controls. Trend towards more pedometer steps in intervention group at 12 months compared to controls. Intervention group with greater increase in pedometer steps from 6 to 12 and 0 to 12 months than controls. Trend towards greater change in activity from 6 to12 and 0 to12 months in intervention group than controls. Activity at baseline and 6 months (self-reported and pedometer) not statistically different between both groups.
Winter [24]	Accelerometer attached to the ankle Number of minutes a patient performed more than 50 gait cycles/min was analyzed (moderate and high level).	Constant increase in volume of activity in intervention and control group from each time point to the next. In both groups, significant improvement comparing post-treatment measurements to 6 weeks after surgery. Trend towards greater volume of physical activity in intervention group than control at each measurement. During treatment, patients rarely performed activity on moderate or higher level. Moderate activities slightly increased after cessation of treatment. Greater, non-significant increase in moderate physical activity in intervention group.
Wurz [33]	Leisure Score Index (LSI) of the GLTEQ	No significant differences during intervention in frequency patients engaged in physical activity. Significant increases in duration patients spent physically active. Patients reported significant increases in time spent in mild, moderate, and strenuous physical activity. Significant increase in total physical activity (MET hours/week).

* only outcomes assessed by Actigraph accelerometer, GSLTPAQ, HBSCQ, ACTIVITYGRAM questionnaire shown; all results shown in supplementary material. ° results shown for all cancer diagnoses combined; separate results for leukemia, lymphoma and solid tumor patients in supplementary material. ^‡^ Data on screentime (screen, playing games, use of computer, tablet or smartphone) available in Supplementary Material.

**Table 7:** Results of included studies reporting on muscle strength.

Author	How Outcome Assesseed	Result Overview
Braam [15]	Upper body muscle strength: highest score of shoulder, elbow and grip strength combined Lower body muscle strength: sum of the highest hip, knee and ankle-dorsiflexion scores	At 4 months, no significant differences between intervention and control group in upper and lower body muscle strength; trend towards decrease in upper and increase in lower body muscle strength in the intervention group. At 12 months significant improvement in lower body muscle strength and trend towards improvement in upper body muscle strength in intervention group compared to control group. Upper and lower body muscle strength significantly increased in intervention group over time.
Cox [16]	Hand grip strength, knee extension, ankle dorsiflexion strength	For all three outcomes, no significant difference in the change over time (baseline vs. ~135 weeks) between intervention and control group.
Fiuza-Luces [17]	5-repetition maximum(5-RM) with bench press, lateral row, and leg press	Significant group–time interaction effect for all the tests comparing intervention and control group. Performance significantly increased in intervention group following training compared to baseline. Trend towards decrease with detraining in intervention group, but detraining values still tended to be higher compared with baseline for leg and bench press.
Fridh [18]	Hand grip strength by hand dynamometer	Trend towards higher handgrip strength in intervention compared to control group in both hands.
Marchese [27]	Knee extension strength and ankle dorsiflexion strength	Knee extension strength significantly increased in intervention group from baseline to 12 weeks; stable in control. No significant differences between groups for ankle dorsiflexion strength from baseline to 12 weeks. Ankle dorsiflexion strength was significantly lower in intervention and control group compared to normal values. Knee extension strength significantly lower in both groups than normal values at baseline. Knee extension strength remained significantly lower in control group at 12 weeks, but increased to normal range in intervention group
Nielsen [20]	Handgrip strength by hand dynamometer	No difference in changes over time in right and left handgrip strength between intervention and control group, but intervention group had better results at all time points. Intervention and control were significantly worse than healthy children in right and left handgrip strength at baseline.
Perondi [21]	10-RM with bench press, lateral pull down, leg press and leg extension	Significant improvement in all four outcomes from baseline to 12 weeks following the intervention.
San Juan [22]	6-RM of upper (seated bench press and seated lateral row) and lower body (leg press)	All three outcomes significantly improved from baseline to 8 weeks following the intervention.
Vriens **°** [28]	Quadriceps and tibialis anterior strength	At all timepoints, patients’ quadriceps strength was significantly lower compared to healthy peers. Significant change in quadriceps strength during treatment: strongest decrease after induction, then improvement. Significantly lower tibialis anterior strength in patients compared to healthy peers at week 5 and 78 with recovery between week 5 and 22. Overall, no significant change over time.

° only results from whole cohort shown in table; stratified by age in Supplementary Material.

**Table 8:** Results of included studies reporting on cardiopulmonary fitness.

Author	How Outcome Was Assesseed	Main Results of VO_2_peak Only *
Braam [15]	CPET (Godfrey protocol): VO_2_peak	At 4 months and 12 months, no significant differences between intervention and control group but trend towards lower VO_2_peak in intervention group. Trend towards improvement in VO_2_peak over time in intervention and control group.
Fiuza-Luces [17]	VO_2_peak	No significant group–time interaction effect for VO_2_peak. Trend towards a training-induced improvement in the ventilatory threshold.
Fridh [18]	CPET (modified Godfrey protocol): VO_2_peak, heart rate, oxygen saturation	Trend towards higher VO_2_peak in the intervention group
Kowaluk [31]	CPET: VO_2_peak, HRpeak, VO_2_, VCO_2_, VE, VE/VCO_2_, MET, test duration	Mean VO_2_peak, at baseline and after 14 months not significantly different between intervention and control group; trend towards higher VO_2_peak in intervention group. Significant improvement in VO_2_peak in the intervention group from baseline to 14 months follow-up; no significant improvement in the control group.
Nielsen [20]	CPET: VO_2_peak, Watt	Significantly higher VO_2_peak in intervention compared to control group over time. Intervention group performed significantly better 6 months after diagnosis compared to control group. Trend to decrease in VO_2_peak control group over time.
Thorsteinsson [23]	VO_2_peak	Children with cancer had significantly lower VO_2_peak than age- and gender-matched controls at every time point.

* all results shown in Supplementary Material.

**Table 9:** Results of included studies reporting on other outcomes.

Author	Outcome Assessment	Main Results of Ankle Range of Motion (ROM) *
**Flexibility**		
Cox [16]	Active and passive ankle dorsiflexion by goniomegtry	Active and passive ancle dorsiflexion decreased in both groups over time and on both sides. Control group scored only significantly better on left active ankle dorsiflexion at T3 than the intervention group.
Hartman [26]	Passive ankle dorsiflexion by goniomegtry	Passive ankle dorsiflexion of both groups combined decreases significantly from diagnosis to cessation of treatment. No significant difference in decrease in passive dorsiflexion over time between intervention and control group. Five children in intervention group needed night splints to maintain ankle dorsiflexion mobility, none in control group.
Marchese [27]	Active ankle dorsiflexion range of motion (ROM)	Significant increases in active ankle dorsiflexion ROM in intervention group, remained stable in control group between pre- and post-test assessments.
Wurz [33]	Hamstring-flexibility (Back-Saver Sit and Reach Test). Passive and active ankle ROM assessed by goniometry	ROM did not significantly change over time, with a trend towards a decrease with passive more than active ROM. Hamstring flexibility improved significantly on both sides over time.
**Motor performance**		
Cox [16]	Bruininks-Oseretsky Test of Motor Proficiency Short Form (BOTSF-2)	No significant difference in BOTSF-2 over time (baseline vs. 135 weeks) between the intervention and the control group.
Hamari [30]	Movement-Assessment Battery for Children 2 (M-ABC2)	The performance decreased in both groups over time and the decrease in the median M-ABC2 scores over time did not differ significantly between the groups.
Hartman [26]	Children < 3.5 years: Dutch Bayley Scales of Infant Development (BSID-II) Children > 4 years: Dutch Movement assessment Battery for Children	At diagnosis motor performance of patients was significantly impaired compared to healthy peers. Trend towards improvement in motor performance from diagnosis to end of treatment in both groups combined. No significant difference between the intervention and control group in change in motor performance over time
**Additional aspects**		
Munsie [19]	Common Terminology Criteria for Adverse Events (CTCAE): fatigue, nausea, pain, mood disturbance, diarrhoe, constipation, vomiting, peripheral neuropathy, dyspnoe, mucositis, insomnia	Control group reported significantly more severe fatigue (≥Grade 3) than intervention group; no significant differences for all other outcomes. Increase in fatigue between week 1 and week 10 in control group and decrease in intervention group (descriptive only). Significant increase in incidence of severe fatigue in control group compared to intervention group. Mean grade of nausea decreased for both groups from week 1 to week 10 (descriptive). Control group reported Grade 3 nausea in weeks 3, 5 and 6, an no Grade 3 reporing in intervention group. Mean pain toxicity over time was lower in intervention group compared to controls (descriptive only). Mean mood disturbance toxicity increased over time in both groups (descriptive only). More Grade 3 toxicity mood disturbance in control group over time compared to intervention group (descriptive only)

* all results shown in Supplementary Material.

## Supplementary Materials

The following supporting information can be downloaded at https://www.mdpi.com/article/10.3390/curroncol32040234/s1, Table S1: Search strategy; Table S2: Quality assessment; Excel S1: comprehensive overview over all outcomes, including separate sheets on “Characteristics”, “Intervention Outcome1”, “Intervention Outcome2”, “Cardiopulmonary fitness”, “Physical performance”, “Motor performance”, “Physical activity”, “Muscle strength”, “QoL”, “Balance and coordination”, and “Flexibility”.

## Abbreviations

The following abbreviations are used in this manuscript:

ALL	Acute lymphoblastic leukemia
CNS	Central nervous system
CPET	Cardiopulmonary exercise test
CPM	Counts per minute
GLTEQ	Godin-Leisure-Time Exercise Questionnaire
GCLTPAQ	Godin-Shepard leisure time physical activity questionnaire
HBSCQ	Health Behaviour in School-Aged Children Questionnaire
MET	Metabolic equivalent
MVPA	Moderate to Vigorous Physical Activity
PA	Physical activity
PedsQL	Pediatric Quality of life Inventory;
QoL	Quality of life
RCT	Randomized controlled trial
ROM	Range of motion
TUDS	Timed Up and Down Stairs test
TUG	Timed Up and Go tests
VO_2_peak	Peak oxygen uptake
6-MWT	6 min walk test
5-RM 10-RM	Maximum strength capacity to perform five/ten repetitions until muscular exhaustion/fatigue

## References

[1] Ward Z.J., Yeh J.M., Bhakta N., Frazier A.L., Girardi F., Atun R. (2019). Global childhood cancer survival estimates and priority-setting: A simulation-based analysis. Lancet Oncol..

[2] Erdmann F., Frederiksen L.E., Bonaventure A., Mader L., Hasle H., Robison L.L., Winther J.F. (2021). Childhood cancer: Survival, treatment modalities, late effects and improvements over time. Cancer Epidemiol..

[3] American Cancer Society (2024). Cancer in Children.

[4] Hamner T., Latzman R.D., Latzman N.E., Elkin T.D., Majumdar S. (2015). Quality of life among pediatric patients with cancer: Contributions of time since diagnosis and parental chronic stress. Pediatr. Blood Cancer.

[5] Bull F.C., Al-Ansari S.S., Biddle S., Borodulin K., Buman M.P., Cardon G., Carty C., Chaput J.P., Chastin S., Chou R. (2020). World Health Organization 2020 guidelines on physical activity and sedentary behaviour. Br. J. Sports Med..

[6] Grimshaw S.L., Taylor N.F., Conyers R., Shields N. (2022). Promoting positive physical activity behaviors for children and adolescents undergoing acute cancer treatment: Development of the CanMOVE intervention using the Behavior Change Wheel. Front. Pediatr..

[7] Langworthy E., Gokal K., Kettle V.E., Daley A.J. (2023). Effects of physical activity interventions on physical activity and health outcomes in young people during treatment for cancer: A systematic review and meta-analysis of randomised controlled trials. BMJ Open Sport. Exerc. Med..

[8] Rapti C., Dinas P.C., Chryssanthopoulos C., Mila A., Philippou A. (2023). Effects of Exercise and Physical Activity Levels on Childhood Cancer: An Umbrella Review. Healthcare.

[9] Braam K.I., van der Torre P., Takken T., Veening M.A., van Dulmen-den Broeder E., Kaspers G.J. (2016). Physical exercise training interventions for children and young adults during and after treatment for childhood cancer. Cochrane Database Syst. Rev..

[10] Baumann F.T., Bloch W., Beulertz J. (2013). Clinical exercise interventions in pediatric oncology: A systematic review. Pediatr. Res..

[11] Morales J.S., Valenzuela P.L., Rincón-Castanedo C., Takken T., Fiuza-Luces C., Santos-Lozano A., Lucia A. (2018). Exercise training in childhood cancer: A systematic review and meta-analysis of randomized controlled trials. Cancer Treat. Rev..

[12] Munsie C., Ebert J., Joske D., Ackland T. (2019). The Benefit of Physical Activity in Adolescent and Young Adult Cancer Patients During and After Treatment: A Systematic Review. J. Adolesc. Young Adult Oncol..

[13] Wurz A., McLaughlin E., Lategan C., Ellis K., Culos-Reed S.N. (2021). Synthesizing the literature on physical activity among children and adolescents affected by cancer: Evidence for the international Pediatric Oncology Exercise Guidelines (iPOEG). Transl. Behav. Med..

[14] Page M.J., McKenzie J.E., Bossuyt P.M., Boutron I., Hoffmann T.C., Mulrow C.D., Shamseer L., Tetzlaff J.M., Akl E.A., Brennan S.E. (2021). The PRISMA 2020 statement: An updated guideline for reporting systematic reviews. BMJ.

[15] Braam K.I., van Dijk-Lokkart E.M., Kaspers G.J.L., Takken T., Huisman J., Buffart L.M., Bierings M.B., Merks J.H.M., van den Heuvel-Eibrink M.M., Veening M.A. (2018). Effects of a combined physical and psychosocial training for children with cancer: A randomized controlled trial. BMC Cancer.

[16] Cox C.L., Zhu L., Kaste S.C., Srivastava K., Barnes L., Nathan P.C., Wells R.J., Ness K.K. (2018). Modifying bone mineral density, physical function, and quality of life in children with acute lymphoblastic leukemia. Pediatr. Blood Cancer.

[17] Fiuza-Luces C., Padilla J.R., Soares-Miranda L., Santana-Sosa E., Quiroga J.V., Santos-Lozano A., Pareja-Galeano H., Sanchis-Gomar F., Lorenzo-González R., Verde Z. (2017). Exercise Intervention in Pediatric Patients with Solid Tumors: The Physical Activity in Pediatric Cancer Trial. Med. Sci. Sports Exerc..

[18] Fridh M.K., Schmidt-Andersen P., Andrés-Jensen L., Thorsteinsson T., Wehner P.S., Hasle H., Schmiegelow K., Larsen H.B. (2023). Children with cancer and their cardiorespiratory fitness and physical function-the long-term effects of a physical activity program during treatment: A multicenter non-randomized controlled trial. J. Cancer Surviv..

[19] Munsie C., Ebert J., Joske D., Collins J., Ackland T. (2024). The potential impact of exercise upon symptom burden in adolescents and young adults undergoing cancer treatment. Support. Care Cancer.

[20] Nielsen M.K.F., Christensen J.F., Frandsen T.L., Thorsteinsson T., Andersen L.B., Christensen K.B., Wehner P.S., Hasle H., Adamsen L., Schmiegelow K. (2020). Effects of a physical activity program from diagnosis on cardiorespiratory fitness in children with cancer: A national non-randomized controlled trial. BMC Med..

[21] Perondi M.B., Gualano B., Artioli G.G., de Salles Painelli V., Filho V.O., Netto G., Muratt M., Roschel H., de Sá Pinto A.L. (2012). Effects of a combined aerobic and strength training program in youth patients with acute lymphoblastic leukemia. J. Sports Sci. Med..

[22] San Juan A.F., Fleck S.J., Chamorro-Viña C., Maté-Muñoz J.L., Moral S., García-Castro J., Ramírez M., Madero L., Lucia A. (2007). Early-phase adaptations to intrahospital training in strength and functional mobility of children with leukemia. J. Strength Cond. Res..

[23] Thorsteinsson T., Larsen H.B., Schmiegelow K., Thing L.F., Krustrup P., Pedersen M.T., Christensen K.B., Mogensen P.R., Helms A.S., Andersen L.B. (2017). Cardiorespiratory fitness and physical function in children with cancer from diagnosis throughout treatment. BMJ Open Sport. Exerc. Med..

[24] Winter C.C., Müller C., Hardes J., Gosheger G., Boos J., Rosenbaum D. (2013). The effect of individualized exercise interventions during treatment in pediatric patients with a malignant bone tumor. Support. Care Cancer.

[25] Yeh C.H., Man Wai J.P., Lin U.S., Chiang Y.C. (2011). A pilot study to examine the feasibility and effects of a home-based aerobic program on reducing fatigue in children with acute lymphoblastic leukemia. Cancer Nurs..

[26] Hartman A., te Winkel M.L., van Beek R.D., de Muinck Keizer-Schrama S.M., Kemper H.C., Hop W.C., van den Heuvel-Eibrink M.M., Pieters R. (2009). A randomized trial investigating an exercise program to prevent reduction of bone mineral density and impairment of motor performance during treatment for childhood acute lymphoblastic leukemia. Pediatr. Blood Cancer.

[27] Marchese V.G., Chiarello L.A., Lange B.J. (2004). Effects of physical therapy intervention for children with acute lymphoblastic leukemia. Pediatr. Blood Cancer.

[28] Vriens A., Verschueren S., Vanrusselt D., Troosters T., Gielis M., Dirix V., Vanderhenst E., Sleurs C., Uyttebroeck A. (2023). Physical fitness throughout chemotherapy in children with acute lymphoblastic leukaemia and lymphoma. Eur. J. Pediatr..

[29] Khodashenas E., Badiee Z., Sohrabi M., Ghassemi A., Hosseinzade V. (2017). The effect of an aerobic exercise program on the quality of life in children with cancer. Turk. J. Pediatr..

[30] Hamari L., Järvelä L.S., Lähteenmäki P.M., Arola M., Axelin A., Vahlberg T., Salanterä S. (2019). The effect of an active video game intervention on physical activity, motor performance, and fatigue in children with cancer: A randomized controlled trial. BMC Res. Notes.

[31] Kowaluk A., Woźniewski M. (2022). Interactive Video Games as a Method to Increase Physical Activity Levels in Children Treated for Leukemia. Healthcare.

[32] Masoud A.E., Shaheen A.A.M., Algabbani M.F., AlEisa E., AlKofide A. (2023). Effectiveness of exergaming in reducing cancer-related fatigue among children with acute lymphoblastic leukemia: A randomized controlled trial. Ann. Med..

[33] Wurz A., Chamorro-Vina C., Guilcher G.M., Schulte F., Culos-Reed S.N. (2014). The feasibility and benefits of a 12-week yoga intervention for pediatric cancer out-patients. Pediatr. Blood Cancer.

[34] Hooke M.C., Hoelscher A., Tanner L.R., Langevin M., Bronas U.G., Maciej A., Mathiason M.A. (2019). Kids Are Moving: A Physical Activity Program for Children With Cancer. J. Pediatr. Oncol. Nurs..

[35] Moyer-Mileur L.J., Ransdell L., Bruggers C.S. (2009). Fitness of children with standard-risk acute lymphoblastic leukemia during maintenance therapy: Response to a home-based exercise and nutrition program. J. Pediatr. Hematol. Oncol..

[36] Grimshaw S.L., Taylor N.F., Shields N. (2016). The Feasibility of Physical Activity Interventions During the Intense Treatment Phase for Children and Adolescents with Cancer: A Systematic Review. Pediatr. Blood Cancer.

[37] Götte M., Söntgerath R., Gauß G., Wiskemann J., Buždon M., Kesting S. (2022). A National Implementation Approach for Exercise as Usual Care in Pediatric and Adolescent Oncology: Network ActiveOncoKids. Pediatr. Exerc. Sci..

[38] Götte M., Gauß G., Dirksen U., Driever P.H., Basu O., Baumann F.T., Wiskemann J., Boos J., Kesting S.V. (2022). Multidisciplinary Network ActiveOncoKids guidelines for providing movement and exercise in pediatric oncology: Consensus-based recommendations. Pediatr. Blood Cancer.

[39] Götte M., Kesting S., Winter C., Rosenbaum D., Boos J. (2014). Experience of barriers and motivations for physical activities and exercise during treatment of pediatric patients with cancer. Pediatr. Blood Cancer.

[40] Caru M., Duhamel G., Marcil V., Sultan S., Meloche C., Bouchard I., Drouin S., Bertout L., Laverdiere C., Sinnett D. (2020). The VIE study: Feasibility of a physical activity intervention in a multidisciplinary program in children with cancer. Support. Care Cancer.

[41] Saultier P., Vallet C., Sotteau F., Hamidou Z., Gentet J.C., Barlogis V., Curtillet C., Verschuur A., Revon-Riviere G., Galambrun C. (2021). A Randomized Trial of Physical Activity in Children and Adolescents with Cancer. Cancers.

[42] Kesting S., Weeber P., Schönfelder M., Pfluger A., Wackerhage H., von Luettichau I. (2022). A Bout of High-Intensity Interval Training (HIIT) in Children and Adolescents during Acute Cancer Treatment-A Pilot Feasibility Study. Cancers.

