# Supplementary material for: Supervised Physical Activity Interventions in Children and Adolescents with Cancer Undergoing Treatment—A Systematic Review
Source: Curr Oncol. 2025 Apr 17;32(4):234. doi: 10.3390/curroncol32040234 (PMC12025492; doi:10.3390/curroncol32040234)
Supplement: Supplementary file 1 [file curroncol-32-00234-s001.zip › PhysicaActivity_Review_Supplement.pdf]

Supervised Physical Activity Interventions in Children and Adolescents with Cancer Undergoing Treatment—A Systematic Review

Nadja Battanta, Krystyna Lange, Sabine V. Kesting, Daniela Marx-Berger, Philip Heesen, Hannah Ober, Aron Onerup, Saskia M.F. Pluijm, Eva Scheler, Emma J. Verwaaijen, Katrin Scheinemann and Maria Otth

Supplemental Table S1: Search Strategy.

**#1 Children**	infant[Title/Abstract] OR infan*[Title/Abstract] OR newborn[Title/Abstract] OR newborn*[Title/Abstract] OR new-born*[Title/Abstract] OR baby[Title/Abstract] OR baby*[Title/Abstract] OR babies[Title/Abstract] OR neonat*[Title/Abstract] OR perinat*[Title/Abstract] OR postnat*[Title/Abstract] OR child[Title/Abstract] OR child*[Title/Abstract] OR schoolchild*[Title/Abstract] OR schoolchild[Title/Abstract] OR school child[Title/Abstract] OR school child*[Title/Abstract] OR kid[Title/Abstract] OR kids[Title/Abstract] OR toddler*[Title/Abstract] OR adolescent[Title/Abstract] OR adolescen*[Title/Abstract] OR adoles*[Title/Abstract] OR teen*[Title/Abstract] OR boy*[Title/Abstract] OR girl*[Title/Abstract] OR minors[Title/Abstract] OR minors*[Title/Abstract] OR underag*[Title/Abstract] OR under ag*[Title/Abstract] OR juvenil*[Title/Abstract] OR youth*[Title/Abstract] OR kindergar*[Title/Abstract] OR puberty[Title/Abstract] OR puber*[Title/Abstract] OR pubescen*[Title/Abstract] OR prepubescen*[Title/Abstract] OR prepuberty*[Title/Abstract] OR pediatrics[Title/Abstract] OR pediatric*[Title/Abstract] OR paediatric*[Title/Abstract] OR peadiatric*[Title/Abstract] OR school*[Title/Abstract] OR nursery school*[Title/Abstract] OR preschool*[Title/Abstract] OR pre school*[Title/Abstract] OR primary school*[Title/Abstract] OR secondary school*[Title/Abstract] OR elementary school*[Title/Abstract] OR elementary school[Title/Abstract] OR high school*[Title/Abstract] OR highschool*[Title/Abstract] OR school age[Title/Abstract] OR schoolage[Title/Abstract] OR school age*[Title/Abstract] OR schoolage*[Title/Abstract] OR infancy[Title/Abstract] OR schools, nursery[Title/Abstract] OR infant, newborn[Title/Abstract]
**#2 Cancer and childhood cancer**	cancer[Title/Abstract] OR oncology[Title/Abstract] OR oncolog*[Title/Abstract] OR neoplasms[Title/Abstract] OR neoplas*[Title/Abstract] OR carcinoma[Title/Abstract] OR carcinom*[Title/Abstract] OR tumor[Title/Abstract] OR tumour[Title/Abstract] OR tumor*[Title/Abstract] OR tumour*[Title/Abstract] OR cancer*[Title/Abstract] OR malignan*[Title/Abstract] OR hematooncological[Title/Abstract] OR hemato oncological[Title/Abstract] OR hemato-oncological hemato oncological[Title/Abstract] OR hematologic neoplasms hemato oncological[Title/Abstract] OR hematolo* hemato oncological[Title/Abstract] OR bone marrow transplantation hemato oncological[Title/Abstract] OR bone marrow transplant* bone marrow transplantation hemato oncological[Title/Abstract] OR pediatric oncology[Title/Abstract] OR paediatric oncology[Title/Abstract] OR pediatric oncology[Title/Abstract] OR childhood cancer[Title/Abstract] OR childhood tumor[Title/Abstract] OR childhood tumors[Title/Abstract] OR leukemia[Title/Abstract] OR leukemi*[Title/Abstract] OR leukaemi*[Title/Abstract] OR "childhood ALL"[Title/Abstract] OR AML[Title/Abstract] OR (leukemia, lymphocytic, acute[mh]) OR leukemia, lymphocytic, acute*[Title/Abstract] OR lymphoma[Title/Abstract] OR lymphom*[Title/Abstract] OR hodgkin[Title/Abstract] OR hodgkin*[Title/Abstract] OR T-cell[Title/Abstract] OR B-cell[Title/Abstract] OR non-hodgkin[Title/Abstract] OR non-hodgkin*[Title/Abstract] OR sarcoma[Title/Abstract] OR sarcom*[Title/Abstract] OR sarcoma, Ewing's[Title/Abstract] OR Ewing*[Title/Abstract] OR osteosarcoma sarcoma, Ewing's[Title/Abstract] OR osteosarcom*[Title/Abstract] OR wilms tumor[Title/Abstract] OR wilms*[Title/Abstract] OR nephroblastom*[Title/Abstract] OR neuroblastoma[Title/Abstract] OR neuroblastom*[Title/Abstract] OR rhabdomyosarcoma[Title/Abstract] OR rhabdomyosarcom*[Title/Abstract] OR teratoma[Title/Abstract] OR teratom*[Title/Abstract] OR hepatoma[Title/Abstract] OR hepatom*[Title/Abstract] OR hepatoblastoma[Title/Abstract] OR hepatoblastom*[Title/Abstract] OR PNET[Title/Abstract] OR medulloblastoma[Title/Abstract] OR medulloblastom*[Title/Abstract] OR PNET*[Title/Abstract] OR (neuroectodermal tumors, primitive)[Title/Abstract] OR retinoblastoma[Title/Abstract] OR retinoblastom*[Title/Abstract] OR meningioma[Title/Abstract] OR meningiom*[Title/Abstract] OR glioma[Title/Abstract] OR gliom*[Title/Abstract] OR brain tumor*[Title/Abstract] OR brain tumour*[Title/Abstract] OR brain cancer*[Title/Abstract] OR brain neoplasm*[Title/Abstract] OR intracranial neoplasm*[Title/Abstract] OR central nervous system neoplasm[Title/Abstract] OR central nervous system neoplasms[Title/Abstract] OR central nervous system neoplasm*[Title/Abstract] OR central nervous system tumor[Title/Abstract] OR central nervous system tumour[Title/Abstract] OR central nervous system tumor*[Title/Abstract] OR central nervous system tumour*[Title/Abstract] OR pediatric oncology[Title/Abstract] OR paediatric oncology[Title/Abstract] OR childhood cancer[Title/Abstract] OR childhood tumor[Title/Abstract] OR childhood tumors[Title/Abstract] OR childhood tumour[Title/Abstract] OR childhood tumours[Title/Abstract] OR childhood tumor*[Title/Abstract] OR childhood tumour[Title/Abstract] OR (leukemia, lymphocytic, acute[mh])
**#3 Physical exercise training therapy**	exercise[Title/Abstract] OR exercises[Title/Abstract] OR Exercises[MeSH] OR exercis*[Title/Abstract] OR Exercise, Physical[MeSH] OR Exercises, Physical[MeSH] OR Physical Exercise[MeSH] OR Physical Exercises[MeSH] OR Physical Exercis*[Title/Abstract] OR physical exercise OR[Title/Abstract] Exercise, Isometric[MeSH] OR Exercises, Isometric[MeSH] OR Isometric Exercises[MeSH] OR Isometric Exercise[MeSH] OR Exercise, Aerobic[MeSH] OR Aerobic Exercises[MeSH] OR Exercises, Aerobic[MeSH] OR Aerobic Exercise[MeSH] OR exercise therapy[Title/Abstract] OR Therapy, Exercise[MeSH] OR Exercise Therapies[MeSH] OR Therapies, Exercise[MeSH] OR exercise therapy[Title/Abstract] OR exercise therapies OR Warm-Up Exercise[MeSH] OR Exercise, Warm-Up[MeSH] OR Exercises, Warm-Up[MeSH] OR Warm-Up Exercise[MeSH] OR Modalities, Physical Therapy[MeSH] OR Modality, Physical Therapy[MeSH] OR Physical Therapy Modality[MeSH] OR Physiotherapy Techniques[MeSH] OR Physiotherapies Techniques[MeSH] OR Physical Therapy Techniques[MeSH] OR Physical Therapy Technique[MeSH] OR Techniques, Physical Therapy[MeSH] OR Physical Therapy[MeSH] OR Physical Therapies[MeSH] OR Therapy, Physical[MeSH] OR Neurological Physiotherapy[MeSH] OR Physiotherapy, Neurological[MeSH] OR physiotherapy[Title/Abstract] OR physiotherap*[Title/Abstract] OR stability training[Title/Abstract] OR training*[Title/Abstract] OR muscle stretching exercise[MeSH] OR muscle stretching exercise*[Title/Abstract] OR physical therapy[Title/Abstract]

	OR physical therapies[Title/Abstract] OR stretch*[Title/Abstract] OR Exercise Movement Technics[MeSH] OR exercise movement technique*[Title/Abstract] OR Movement Techniques, Exercise[MeSH] OR exercise movement techn*[Title/Abstract] OR pilatesbased exercise[Title/Abstract] OR pilates-based exercise*[Title/Abstract] OR Pilates Based Exercises[MeSH] OR Pilates-Based Exercises[MeSH] OR Exercises, Pilates-Based[MeSH] OR pilates[Title/Abstract] OR Pilates Training[MeSH] OR Training, Pilates[MeSH] OR gymnastics[Title/Abstract] OR gymnastic[Title/Abstract] OR gymnastic*[Title/Abstract] OR swimming[Title/Abstract] OR swim[Title/Abstract] OR locomotion[MeSH] OR locomotion*[Title/Abstract] OR treadmill[Title/Abstract] OR walking[Title/Abstract] OR Walking[MeSH] OR walk[Title/Abstract] OR running[Title/Abstract] OR Running[MeSH] OR cycling[Title/Abstract] OR jogging[Title/Abstract] OR occupational therapy[Title/Abstract] OR Occupational Therapy[MeSH] OR occupational therapies[Title/Abstract] OR functional therapy[text] OR functional therapies[text] OR training program[Title/Abstract] OR fitness[Title/Abstract] OR cardio training[Title/Abstract] OR weight lifting[Title/Abstract] OR power training[Title/Abstract] OR muscle training[Title/Abstract] OR rowing[Title/Abstract] OR Sport[MeSH] OR sport*[Title/Abstract] OR jump[Title/Abstract] OR jumping[Title/Abstract] OR physical activity[Title/Abstract] OR Physical Activity[MeSH] OR physical activities OR physical activ* OR Activities, Physical[MesH] OR Activity, Physical[MesH] OR Physical Activities[MesH] OR exercise training[Title/Abstract] OR exercise train*[Title/Abstract] OR Exercise Training[MesH] OR Exercise Trainings[MesH] OR Training, Exercise[MesH] OR Trainings, Exercise[MesH] OR athletic*[Title/Abstract] OR motor activity[Title/Abstract] OR motor activities[Title/Abstract] OR motor activity[MeSH] OR resistance training[MeSH] OR resistance training[Title/Abstract] OR resistance train*[Title/Abstract] OR strength training[Title/Abstract] OR Strengths Training[MeSH] OR strength train* OR exercise program[Title/Abstract] OR exercise programs[Title/Abstract] OR exercise program*[Title/Abstract] OR accelerometry[MeSH] OR accelerometry[Title/Abstract] OR accelerometr*[Title/Abstract] OR exergaming[MeSH] OR exergam*[Title/Abstract] OR exergame[Title/Abstract] OR exergames[Title/Abstract] OR active-video gaming[Title/Abstract] OR active-video game*[Title/Abstract] OR active-video gami*[Title/Abstract] OR active video gaming[Title/Abstract] OR active video game*[Title/Abstract] OR active video gami*[Title/Abstract] OR pedometer[Title/Abstract] OR pedometers[Title/Abstract] OR pedomet*[Title/Abstract] OR occupational activities[Title/Abstract] OR occupational activity[Title/Abstract] OR occupational activ*[Title/Abstract] OR yoga[Title/Abstract]
**#4 During treatment**	active treatment[Title/Abstract] OR during treatment[Title/Abstract] OR under treatment[Title/Abstract] OR undergoing treatment[Title/Abstract]
	#1 AND #2 AND #3 AND #4

Supplemental Table S2: Quality Assessment.

**Randomized controlled trials (1)**					
	**Braam**	**Cox**	**Hartmann**	**Kowaluk**	**Munsie**
Was true randomization used for assignment of participants to treatment groups?	Yes	Yes	Yes	Unclear	Yes
Was allocation to treatment groups concealed?	Yes	Yes	Yes	Unclear	Yes
Were treatment groups similar at the baseline?	Yes	Yes	Yes	Yes	Yes
Were participants blind to treatment assignment?	Uncl. (NS)	Uncl. (NS)	Uncl. (NS)	Uncl. (NS)	Uncl. (NS)
Were those delivering treatment blind to treatment assignment?	Uncl. (NS)	Uncl. (NS)	Uncl. (NS)	Uncl. (NS)	Uncl. (NS)
Were outcomes assessors blind to treatment assignment?	Uncl.	Yes	Yes	Unclear	Yes
Were treatment groups treated identically other than the intervention of interest?	Yes	Yes	Yes	Yes	Yes
Was follow up complete and if not, were differences between groups in terms of their follow up adequately described and analyzed?	Yes	Yes	Yes	Unclear	Unclear
Were participants analyzed in the groups to which they were randomized?	Yes	Yes	Yes	Yes	Yes
Were outcomes measured in the same way for treatment groups?	Yes	Yes	Yes	Yes	Yes
Were outcomes measured in a reliable way?	Yes	Yes	Yes	Yes	Yes
Was appropriate statistical analysis used?	Yes	Yes	Yes	Yes	Yes
Was the trial design appropriate, and any deviations from the standard RCT design (individual randomization, parallel groups) accounted for in the conduct and analysis of the trial?	Yes	Yes	Yes	Yes	Yes
**Total (yes)**	10/13	11/13	11/13	7/13	10/13
**Randomized controlled trials (2)**					
	**Fiuza-Luces**	**Hamari**	**Marchese**	**Masoud**	**Moyer-Mileur**
Was true randomization used for assignment of participants to treatment groups?	Yes	Yes	Yes	No	Unclear
Was allocation to treatment groups concealed?	Unclear	Yes	No	Unclear	Unclear
Were treatment groups similar at the baseline?	Yes	Yes	Yes	Yes	Yes
Were participants blind to treatment assignment?	Uncl. (NS)	Uncl. (NS)	Uncl. (NS)	Uncl. (NS)	Uncl. (NS)
Were those delivering treatment blind to treatment assignment?	Uncl. (NS)	Uncl. (NS)	Uncl. (NS)	Uncl. (NS)	Uncl. (NS)
Were outcomes assessors blind to treatment assignment?	Yes	Yes	No	Yes	Unclear
Were treatment groups treated identically other than the intervention of interest?	Yes	Yes	Yes	Yes	Yes
Was follow up complete and if not, were differences between groups in terms of their follow up adequately described and analyzed?	Yes	Unclear	Unclear	Yes	Unclear
Were participants analyzed in the groups to which they were randomized?	Yes	Yes	Yes	Yes	Yes
Were outcomes measured in the same way for treatment groups?	Yes	Yes	Yes	Yes	Yes
Were outcomes measured in a reliable way?	Yes	Yes	Yes	Yes	Yes
Was appropriate statistical analysis used?	Yes	Yes	Yes	Yes	Yes
Was the trial design appropriate, and any deviations from the standard RCT design (individual randomization, parallel groups) accounted for in the conduct and analysis of the trial?	Yes	Yes	Yes	Unclear	Unclear
**Total (yes)**	10/13	10/13	8/13	8/13	6/13
**Quasi experimental studies (1)**					
	**Fridh**	**Hooke**	**Khodas-henas**	**Nielsen**	**Perondi**
Is it clear in the study what is the "cause" and what is the "effect" (i.e. there is no confusion about which variable comes first)?	Yes	Yes	Yes	Yes	Yes
Was there a control group?	Yes	Yes	Yes	Yes	Yes
Were participants included in any comparisons similar?	Yes	Yes	Unclear	Yes	Yes

Were the participants included in any comparisons receiving similar treatment/care, other than the exposure or intervention of interest?	Yes	Yes	Yes	Yes	Yes
Were there multiple measurements of the outcome, both pre and post the intervention/exposure?	No (not P&P)	No (not P&P)	Yes	Yes	Yes
Were the outcomes of participants included in any comparisons measured in the same way?	Yes	Yes	Yes	Yes	Yes
Were outcomes measured in a reliable way?	Yes	Yes	Yes	Yes	Yes
Was follow-up complete and if not, were differences between groups in terms of their follow-up adequately described and analyzed?	Unclear	Unclear	Unclear	Yes	Yes
Was appropriate statistical analysis used?	Yes	Yes	Yes	Yes	Yes
**Total (yes)**	7/9	7/9	7/9	9/9	9/9
**Quasi experimental studies (2)**					
	**San Juan**	**Thorstein-sson**	**Winter**	**Wurz**	**Yeh**
Is it clear in the study what is the “cause” and what is the “effect” (i.e. there is no confusion about which variable comes first)?	Yes	Yes	Yes	Yes	Yes
Was there a control group?	Yes	Yes	Yes	Yes	Yes
Were participants included in any comparisons similar?	Yes	Yes	Yes	Yes	Yes
Were the participants included in any comparisons receiving similar treatment/care, other than the exposure or intervention of interest?	Yes	Yes	Yes	Yes	Yes
Were there multiple measurements of the outcome, both pre and post the intervention/exposure?	Unclear	Yes	Yes	Yes	Yes
Were the outcomes of participants included in any comparisons measured in the same way?	Yes	Yes	Yes	Yes	Yes
Were outcomes measured in a reliable way?	Yes	Yes	Yes	Yes	Yes
Was follow-up complete and if not, were differences between groups in terms of their follow-up adequately described and analyzed?	Unclear	Yes	No	Yes	Unclear
Was appropriate statistical analysis used?	Yes	Yes	Yes	Yes	Yes
**Total (yes)**	7/9	9/9	8/9	9/9	8/9
**Cohort study**					
	**Vriens**				
Were the two groups similar and recruited from the same population?	not applicable				
Were the exposures measured similarly to assign people to both exposed and unexposed groups?	not applicable				
Was the exposure measured in a valid and reliable way?	Yes				
Were confounding factors identified?	not applicable				
Were strategies to deal with confounding factors stated?	not applicable				
Were the groups/participants free of the outcome at the start of the study (or at the moment of exposure)?	Yes				
Were the outcomes measured in a valid and reliable way?	Yes				
Was the follow up time reported and sufficient to be long enough for outcomes to occur?	Yes				
Was follow up complete, and if not, were the reasons to loss to follow up described and explored?	Yes				
Were strategies to address incomplete follow up utilized?	Yes				
Was appropriate statistical analysis used?	Yes				
**Total (yes)**	7/11 (7/7)				

NS, not stated ; P&P, pre and post
